# Neuropathy in COVID-19 associated with dysbiosis-related inflammation

**DOI:** 10.3906/biy-2105-53

**Published:** 2021-08-30

**Authors:** Busra AKTAS, Belma ASLIM

**Affiliations:** 1 Department of Molecular Biology and Genetics, Faculty of Arts and Sciences, Burdur Mehmet Akif Ersoy University, Burdur Turkey; 2 Department of Biology, Faculty of Sciences, Gazi University, Ankara Turkey

**Keywords:** COVID-19, gut microbiota dysbiosis, neuropathy, inflammation, gut–lung axis, gut–brain axis

## Abstract

Although COVID-19 affects mainly lungs with a hyperactive and imbalanced immune response, gastrointestinal and neurological symptoms such as diarrhea and neuropathic pains have been described as well in patients with COVID-19. Studies indicate that gut–lung axis maintains host homeostasis and disease development with the association of immune system, and gut microbiota is involved in the COVID-19 severity in patients with extrapulmonary conditions. Gut microbiota dysbiosis impairs the gut permeability resulting in translocation of gut microbes and their metabolites into the circulatory system and induce systemic inflammation which, in turn, can affect distal organs such as the brain. Moreover, gut microbiota maintains the availability of tryptophan for kynurenine pathway, which is important for both central nervous and gastrointestinal system in regulating inflammation. SARS-CoV-2 infection disturbs the gut microbiota and leads to immune dysfunction with generalized inflammation. It has been known that cytokines and microbial products crossing the blood-brain barrier induce the neuroinflammation, which contributes to the pathophysiology of neurodegenerative diseases including neuropathies. Therefore, we believe that both gut–lung and gut–brain axes are involved in COVID-19 severity and extrapulmonary complications. Furthermore, gut microbial dysbiosis could be the reason of the neurologic complications seen in severe COVID-19 patients with the association of dysbiosis-related neuroinflammation. This review will provide valuable insights into the role of gut microbiota dysbiosis and dysbiosis-related inflammation on the neuropathy in COVID-19 patients and the disease severity.

## 1. Introduction

Over a hundred million people around the world have been affected by the recent outbreak of the novel coronavirus (SARS-CoV-2), COVID-19^1^
Worldometers (2021). Coronavirus cases [online]. Website https://www.worldometers.info/coronavirus [accessed 20 May 2021].. Some of the individuals infected with SARS-CoV-2 either remain asymptomatic or have mild symptoms while some of them are admitted to intensive care unit (ICU) with severe symptoms and develop severe acute respiratory distress syndrome (ARDS) (Chen et al., 2020b). SARS-CoV-2 infection accompanied by multiple organ failure syndrome mostly results in death due to damage in vital organs (Farooqui, 2021). SARS-CoV-2 has known to affect mainly the respiratory system or lungs; nonetheless, symptoms associated with gastrointestinal (GI) system have been described as well in the patients with COVID-19. Fever, cough, sore throat, dyspnea, and fatigue have been reported as the most prevalent clinical symptoms. Over the past year, GI symptoms emerged as critical manifestations (Schmulson et al., 2021). Vomiting, nausea, and diarrhea have been described as the most prominent GI symptoms in COVID-19. Frequency of GI symptoms in COVID-19 patients ranges from 5% to 80% with diarrhea ranging from 2% to 50% (Chen et al., 2020a, 2020b; Fortune and Sharaiha, 2020). Interestingly, GI manifestations have been reported as the first symptoms in some cases occurring even before the respiratory symptoms or fever (Song et al., 2020; Lin et al., 2020). It is well established that the digestive system contributes to the COVID-19 illness and gastrointestinal signs at the beginning or during the course of the disease should not be underestimated (Ferreira et al., 2020; Jin et al., 2020; Tao et al., 2020; Wan et al., 2020). While some of the patients display only the GI symptoms, some with GI symptoms, in particular diarrhea, developed severe respiratory complications requiring ventilation support in ICU admission more often relative to those with no GI symptoms (Jin et al., 2020; Pan et al., 2020; Wan et al., 2020). 

Zhang et al. (2021) performed a retrospective study with 409 severe and hospitalized COVID-19 patients and compared inflammatory markers of the patients with diarrhea to those without diarrhea to understand the relationship between gastrointestinal symptoms and immune response in COVID-19 patients. Patients with diarrhea showed a low level of lymphocyte with reduced CD8+ T cells and highly increased tumor necrosis factor-alpha (TNF-α), interleukin-10 (IL-10), and IL-6 levels. The severity of diarrhea with high frequency and long duration was correlated with the inflammatory profile and duration of the course of COVID-19. Moreover, diarrhea was reported more often and more severe among patients who died relative to those survived. Patients with diarrhea tended to develop multiple organ failure and stay in hospital longer. SARS-CoV-2 infection causes lung injury resulting in pneumonia with a hyperactive and imbalanced immune response (Li et al., 2020). Lymphocytopenia and raised level of proinflammatory cytokines and chemokines leading to “cytokine storm” have found to be associated with severe COVID-19 accompanied by severe acute respiratory syndrome in lung and multiple organ dysfunction (Huang et al., 2020; Kalantar-Zadeh et al., 2020; Zheng et al., 2020). 

ARDS and sepsis-induced hypoxic damage, one of the factors causing oxidative stress, lead to neurodegenerative disorders and are associated with structural changes in the brain of the patients with COVID-19 (Dolatshahi et al., 2021). In a large-scale observational study comprising 214 individuals with COVID-19, the ratio of the patients who declared neurologic complaints was about 36% (Mao et al., 2020). Neurologic symptoms reported in COVID-19 patients included headache, vertigo, altered consciousness, encephalitis, neuropathic pain, facial paralysis and olfactory disturbances, loss in sense of taste, cerebrovascular events, and seizure (Bureau et al., 2020; Mao et al., 2020). Although the mechanisms of neurologic damage were unclear in the COVID-19 patients, sepsis, systemic inflammatory response syndrome, and possible SARS-CoV-2 interaction with the angiotensin-converting enzyme 2 (ACE2) receptors in nerves, muscle, and brain were considered to discuss their involvement in the mechanism of action (Mao et al., 2020). Upregulation of the proinflammatory cytokines such as IL-6, IL-8, and TNF-α in COVID-19 patients, particularly in those with more severe disease, raises doubts whether the cause of the severe meningoencephalitis or/and other neurologic manifestations in COVID-19 patients is the peripheral inflammation rather than direct SARS-CoV-2 invasion (Benameur et al., 2020; Dolatshahi et al., 2021). Peripheral cytokines at increased level can cross the blood-brain barrier directly and trigger neuroinflammation, which contributes to the pathophysiology of neurodegenerative diseases including neuropathies (Costello and Dalakas, 2020; Dolatshahi et al., 2021). Genetic predispositions, metabolic risk factors, and dysbiosis in gut microbiota, which are common risk factors for neurodegenerative diseases and COVID-19, are thought to be partly responsible for a higher incidence of neurodegenerative diseases in COVID-19 survivors (Dolatshahi et al., 2021). Since impaired intestinal microbiota decreases the gut epithelial integrity and activate the immune system by molecular mimicry and oxidative stress, gut microbial dysbiosis involves the viral invasion directly into the central nervous system (CNS) as well as the neurodegenerative processes by changing neurotransmission balance.

Gut microbiota communicates with the brain, which is called the gut–brain axis, maintaining homeostasis of the gastrointestinal and the nervous system (Rhee et al., 2009). SARS-CoV-2 affects brain functions most likely through the gut–brain axis associated with dysbiosis and inflammation. Alteration of the microbial composition and its metabolites in the gut is associated with intestinal permeability and systemic immune responses with proinflammatory mediators which may lead to peripheral sensitization of chronic pain in COVID-19 patients (Leclercq et al., 2014; Thevaranjan et al., 2017). Therefore, emerging role of gut microbiota in neuropathic symptoms described by COVID-19 has attracted attention recently. This review highlights the role of the gut microbial dysbiosis on neuropathy in COVID-19 with association of dysbiosis-related inflammation. 

## 2. Gut microbiota in COVID-19 with the impact of medication

Pathophysiology of the digestive manifestations in COVID-19 has not been fully uncovered; however, it is well known that there is a crosstalk between gut microbiota and lung, and growing evidence draws attention to the role of the gut microbiota in COVID-19 severity (Ferreira et al., 2020; Wan et al., 2020; Tao et al., 2020). Microbial populations in the gut contributes to health and influence the homeostatic and physiological functions in humans (Ostaff et al., 2013). While gut microbiota helps in maturation and development of defense system in human body, the immune system constructs the microbiota composition and functions. It is well known that healthy gut microbial composition is altered in various health conditions such as inflammatory bowel disease, arthritis, obesity, type 2 diabetes, and asthma (Aleman and Valenzano 2019; Hufnagl et al., 2020; Tai et al., 2015). Altered gut microbiota to an imbalanced state in healthy individuals, dysbiosis, causes a disruption in balanced immune functionality and a widespread inflammation. When infants are exposed to antibiotics resulting in a shift in the organization of the intestinal microbiota, in the future, their risk of developing inflammatory bowel disease increases due to the impact of gut microbiota on immune development early in life (Shaw et al., 2010; Hviid et al., 2011). Change in the gut microbiota alters the intestinal barrier integrity with its immunomodulatory effect (Leclercq et al., 2014; Thevaranjan et al., 2017). Raised level of inflammation in intestine due to dysbiosis provokes epithelial barrier dysfunction, referred to as leaky gut, and may lead to multiple organ failure with secondary infection (Hanada et al., 2018; Fanos et al., 2020). 

The gut–lung axis maintaining host homeostasis and disease development with immunomodulation is thought to be involved in disease severity and extrapulmonary conditions of COVID-19 with the association of dysbiosis. Damage in the intestinal barrier integrity due to dysbiosis could result in SARS-CoV-2 translocation from lung to gut lumen via circulatory and lymphatic system (Gu et al., 2005; Aktas and Aslim 2020). Conversely, decrease in the gut permeability due to dysbiosis could result in secondary infection by intestinal microorganisms and leads to multiple organ failure (Hanada et al., 2018; Deitch, 2012). Considering the gut–lung crosstalk with the association of gut permeability and systemic inflammation, individuals with gut dysbiosis could be at high risk for severe COVID-19.

Several studies have reported change in microbial composition in feces from patients with COVID-19 (Table). Overall, SARS-CoV-2 infection decreases the diversity and increases the abundance of opportunistic pathogens in the gut. COVID-19 patients from different studies varied in their fecal microbial composition patterns (Table). This variation could be due to life style with different dietary habits among individuals or different treatment procedure applied in different hospitals.

**Table:** Changes in the composition of fecal microbiome of COVID-19 patients.

Modulation of fecal microbiota	*Antibiotics	*Antivirals or other drugs	Geographic location	Gastrointestinal symptoms	References
↑ Ruminococcus gnavus, Eggerthella, Coprobacillus, Lachnospiraceae bacterium, Clostridium ramosum, and Eggerthella lenta ↓ Alistipes sp AP11, Roseburia intestinalis, Burkholderiales bacterium, Eubacterium hallii, Parasutterella excrementihominis, Alistipes indistinctus, Coprobacter fastidiosus, Eubacterium eligens, Bacteriodales bacterium ph8, Bacteroides salyersiae, Odoribacter splanchnicus, Alistipes shahii, Ruminococcus bromii, and Bacteroides massiliensis	None, moxifloxacin, piperacillin/tazobactam, cefuroxime, or levofloxacine	None, lopinavir/ritonavir, arbidol, or ribavirin	Beijing, China	None, diarrhea, constipation, or abdominal distention	(Cao et al. 2021)
↑ Streptococcus, Clostridium, Haemophilus, and Proteobacteria ↓ Prevotella, Akkermansia, Paraprevotella, and Lachnospira	Antibiotics (unspecified)	Antivirals (unspecified), corticosteroids, immunoglobulin, traditional Chinese medicine, probiotics, anticoagulation, or tocilizumab (anti-IL6R)	Hefei, China	Diarrhea, nausea, vomit, anorexia, or abdominal pain	(Tao et al. 2021)
↑ Enterococcus and Enterobacteriaceae ↓ Lactobacillus, Bifidobacterium, Faecalibacterium prausnitzii, Clostridium butyricum, Clostridium leptum, and Eubacterium rectale	Antibiotics (unspecified)	Antifungal drugs or probiotics	NA	NA	** (Tang et al. 2020)
↑ Collinsella aerofaciens, Collinsella tanakaei, Streptococcus infantis, and Morganella morganii ↓ Parabacteroides merdae, Bacteroides stercoris, Alistipes onderdonkii, and Lachnospiraceae bacterium	NA	NA	Hong Kong, China	None or diarrhea	*** (Zuo et al. 2021)
↑ Streptococcus, Rothia, Veillonella, Erysipelatoclostridium, and Actinomyces ↓ Ruminococcaceae,Fusicatenibacter, Anaerostipes, Agathobacter, unclassified Lachnospiraceae, and Eubacterium hallii	NA	NA	Zhejiang, China	None or diarrhea	(S. Gu et al. 2020)
↑ Ruminococcus gnavus, Ruminococcus torques, and Bacteroides dorei ↓ Bifidobacterium adolescentis, Faecalibacterium prausnitzii, and Eubacterium rectale	Antibiotics (unspecified)	None, lopinavir/ritonavir, ribavirin, or oseltamivir. Corticosteroids or proton pump inhibitor	Hong Kong, China	None or diarrhea	(Yeoh et al. 2021)
↑ Clostridium hathewayi, Actinomyces viscosus, and Bacteroides nordii ↓ Eubacterium ventriosum, Faecalibacterium prausnitzii, Roseburia, and Lachnospiraceae.	Amoxycillin, clavulanate cephalosporin, or tetracycline	Lopinavir/ritonavir, or ribavirin. Interferon beta-1b	Hong Kong, China	None or diarrhea	(Zuo et al. 2020)
↑ Streptococcus, Clostridium, Lactobacillus, and Bifidobacterium ↓ Bacteroidetes, Roseburia, Faecalibacterium, Coprococcus, and Parabacteroides.	NA	NA	Anhui, China	NA	(Tao et al. 2020)

Dysbiosis involved in sepsis is one of the reasons for the severity of SARS-CoV-2 infection with the association of the gut–lung axis. Some of the patients with COVID-19 showing gastrointestinal symptoms, in particular diarrhea, could have dysbiotic gut microbiota when they were infected while for others who did not have dysbiotic gut microbiota when infected, the treatment procedure of COVID-19 might cause dysbiosis in their intestinal microbiota. Antibiotics are primary disruptors of gut microbiota breaking the balance between microbiota and immune system and causing metabolic and immunologic changes (Shaw et al., 2010; Hviid et al., 2011). Antibiotic-associated diarrhea is one of the main side effects of antibiotic administration due to the altered organization of the intestinal microbiota (Hickson, 2011). Shift in the gut microbial composition due to antibiotics increases the risk of inflammatory disorders and new infections such as Clostridium difficile infection which generates Clostridium difficile-associated diarrhea (Shaw et al., 2010; Hickson, 2011; Hviid et al., 2011). Antibiotics have been commonly used in COVID-19, particularly to prevent secondary infection, and this extensive antibiotic administration possibly has a role in disruption of the microbial balance leading to diarrhea in COVID-19 patients (Chen et al., 2020b; Guan et al., 2020). The level of depletion in microbial populations is much greater in antibiotic administered patients with COVID-19 (Zuo et al., 2020; Cao et al., 2021;Yeoh et al., 2021). Previously, dysbiosis in mice by antibiotics were found to be associated with viral infections in distal organs (Ichinohe et al., 2011). They reported that antibiotics keep the commensal microbial populations in the gut from regulating immune defense against influenza A virus infection and mice had become more susceptible to the virus infection in lungs. Although there has been no effective treatment for COVID-19, a variety of treatment regimens have been applied to the patients infected by SARS-CoV-2 (Table). Gut microbiota can impact not only the disease symptoms and the severity of the illness but also the drug pharmacokinetics used to treat COVID-19 and patient response to the therapy applied (Noh et al., 2017; Zhang et al., 2018; Zimmermann et al., 2019). When the drug is metabolized by the gut microbiota before absorption, bioavailability of the drugs could be altered as well as the possible side effects. Hydroxychloroquine (HCQ) is an antimalarial drug that has been used for inflammatory disorders including rheumatoid arthritis for years. After the outbreak, it was one of the earliest drugs used for the prevention and treatment of COVID-19 due to its in vitro antiviral effect against SARS- CoV-2 (Yao et al., 2020). It has been demonstrated in mice that HCQ challenge significantly changed the gut microbial diversity with depletion in Firmicutes and increase in Bacteroidetes; however, it did not impact the immune response (Pan et al., 2021). While HCQ have been used alone in some COVID-19 cases, it is sometimes used in combination with azithromycin (Das et al., 2020). Due to adverse outcomes of COVID-19, related to immune response with increased proinflammatory cytokines resulting in cytokine storm, antiinflammatory drugs such as corticosteroids have been used as well (Felsenstein et al., 2020). Additionally, antiviral drugs such as favipiravir, remdesivir, and ribavirin have been commonly used for COVID-19 treatment. The list of the drugs that have been tested as COVID-19 therapeutic agent is much longer than those mentioned here. While some of them are administered alone, some are used in combination depending on the treatment regimen that has been used in clinical practice in different hospitals or different countries. However, in most of the studies, either information about the treatment procedure is limited or treatment regimen covers combination of two or more drugs including HCQ, antiviral drugs, steroids, and antibiotics and varies among patients involved in the same study (Ceccarelli et al., 2021; Cao et al., 2021; Livanos et al., 2021). Different therapeutic interventions can influence the intestinal microbiota and eventually impact the gastrointestinal manifestation in patients with COVID-19 and the severity of the illness associated with dysbiosis during the course of the disease.

## 3. Gut–brain axis in COVID-19

Alternatively, diarrhea or other gastrointestinal manifestations could be the result of the gut-brain axis. Physiological processes of functional gastrointestinal disorders were found to be involved in dysregulation of bidirectional gut–brain interaction in addition to dysbiosis of the gut microbiota with inflammation and impaired gut barrier (Black et al., 2020). Interactions between the gut microbiota and the brain have been explored, and the results indicate that intermediaries resulting from the interaction between the gut immune system and the microbiota may impact the brain functions (Mayer et al., 2014; Shanahan and Quigley, 2014). Changes in the bidirectional relationship between the gut and the nervous system have been shown to play an important role in irritable bowel syndrome (IBS) pathogenesis and associated functional gastrointestinal disorders with GI manifestations such as abdominal pain, diarrhea, or/and constipation (Mari et al., 2020; Simpson et al., 2020; Holvoet et al., 2021). It is interesting that germ-free animals have been found to exaggerate the activation of hypothalamic–pituitary–adrenal axis, a central stress response system, in response to stress. This hyperresponsiveness was reversed by restructuring the microbiota with fecal suspension from animals kept in a pathogen-free environment or through oral inoculation of B. infantis (Sudo et al., 2004; Smith and Vale 2006). Although the exact mechanism is not known fully yet, gut microbiota is capable of impacting stress and visceral hypersensitivity (Moloney et al., 2016). Recent studies performed fecal microbiota transplantation (FMT) from healthy donors to patients with IBS and reported that modulation of the gut microbiota by FMT relieved symptoms such as abdominal pain in IBS patients compared to the control group (Johnsen et al., 2018, 2020; El-Salhy et al., 2021; Holvoet et al., 2021). GI microbiota is a critical piece with its potential to affect neuro-immuno-endocrine pathways. The gut microbiota and its metabolic products are capable of modifying GI functions by impacting the gut barrier integrity, immune function, enteric nervous system (ENS), and brain (Mayer et al., 2014). Studies support that microbiota is involved in adult neural plasticity including microglia activation and neurogenesis and is necessary for normal brain development and healthy brain functions in adulthood (Hsiao et al., 2013; Ogbonnaya et al., 2015; Stilling et al., 2015). Antibiotic-induced microbiota depletion in mice affects cognitive behaviors and anxiety in addition to the gut–brain axis neuromodulators including neuropeptides, monoamines, and tryptophan (Desbonnet et al., 2015). Conversely, the brain can modify the environment of the gut microbiota through ENS, autonomic nervous system, and hypothalamic pituitary axis by altering the luminal secretion, epithelial integrity, mucosal immunity, and release of neurotransmitters (Rhee et al., 2009; Mayer, 2011).

Metabolites produced by gut microbiota such as tryptophan, serotonin, GABA, and short chain fatty acids and immune molecules generated during the immune response against microbes can signal through the intestinal cells and affect the gut locally. Moreover, these molecules can signal through the endocrine and neurocrine system associated cells and impact extraintestinal organs such as the brain (Mayer et al., 2014). Short chain fatty acids produced by intestinal microbiota play an important role in maturation and activation of microglia cells which are macrophage-like immune cells in the CNS (Lin et al., 2020). Microbial products can translocate to the circulatory system from the gut and reach the blood-brain barrier. With the changes in the gut microbial composition, increased intestinal permeability and activated immune response may lead to systemic inflammation and impact the blood-brain barrier, and eventually induce neural injury and neurodegeneration with neuroinflammation (Rhee et al., 2009; Heiss and Olofsson 2019). Furthermore, gastrointestinal tract is the largest endocrine organ in the human body and produce intestinal hormones via the enteroendocrine system with a wide range of targets both intestinal and extraintestinal (Ahlman and Nilsson, 2001). Intestinal hormones, such as neuropeptide Y, glucagon-like peptide 1, and peptide YY, produced by enteroendocrine cells play a role in pain modulation with the association of the immune and nervous systems. It has been shown that gut microbiota communicates these enteroendocrine cells and impact the enteroendocrine metabolism. SARS-CoV-2 infection is thought to result in symptoms of vomiting or nausea through either inducing enteroendocrine release in the gut mucosa or impacting the brainstem directly after getting into the circulatory system (Andrews et al., 2021).

## 4. Gut microbiota and inflammation associated neuropathy in COVID-19

Although SARS-CoV-2 mainly infects the respiratory system, nervous system has been found to be involved in COVID-19 as well, with neurological symptoms including anosmia, headache, anorexia, vertigo, altered consciousness, encephalitis, neuropathic pain, facial paralysis and olfactory disturbances, and loss in sense of taste (Bureau et al., 2020; Mao et al., 2020; Wu et al., 2020). SARS-CoV-2 recognizes the ACE2 receptor and invades the host cell via type II transmembrane serine protease (TMPRSS2) (Zhang et al., 2020). Since the ACE2 receptor and the TMPRSS2 are expressed at relatively low levels in the brain, it is hard to state for now that SARS-CoV-2 directly invades the CNS and causes neurological damage. It has been thought that the gut–brain axis may be involved in COVID-19, and SARS-CoV-2 might affect the CNS via intestine associated inflammation (Shi et al., 2020; Wu et al., 2020). Coronaviruses damage the structure and function of the nervous system with their ability of entering the cerebrospinal fluid (Marasco et al., 2021). Although the mechanism(s) by which SARS-CoV-2 reaches the central nervous system is essentially unknown, it is believed that the blood-brain barrier is involved in the invasion path (Marasco et al., 2021). Previously, neurotropic viruses were shown to infect the ENS continuously and cause GI dysfunction (Brun et al., 2010). 

Decrease in gut barrier function with the change in the gut microbiota due to viral infection will modify the blood-brain barrier integrity and let bacteria, bacterial metabolites, and toxins translocate to the brain via systemic circulation, then will cause damage in brain functions (Lin et al., 2020). Vagus nerve is linked to the neurons in the ENS and carries information from the intestine to the brain; therefore, ENS dysfunction can impact both the gastrointestinal system and brain functionality via gut-brain axis (Tognini, 2017). Recently, Deffner et al. (2020) explored a histological evidence of alternative routes for SARS-CoV-2 neuroinvasion and performed immunostainings for ACE2 and TMPRSS2 in the human ENS and choroid plexus. They reported that enteric neurons and glial cells in the small and large intestine and choroid plexus epithelium cells expressed both ACE2 and TMPRSS2. Studies suggest that SARS-CoV-2 reaching the gut directly or indirectly invades the ENS and travels to the brain via vagus nerve to impact the central nervous system (Jakhmola et al., 2020; Deffner et al., 2020). Moreover, damage in the ENS could cause intestinal dysmotility and disturbed barrier function which lead to translocation of gut microbes and their metabolites into the systemic circulation to the brain, suggesting the bidirectional interaction between gut and the nervous system. On the other hand, coronaviruses are thought to move to the brain via motor or sensory nerve endings along with retrograde neuronal transport through the motor proteins including kinesin and dynein (Cataldi et al., 2020; Marasco et al., 2021). Recently, ENS has been suggested to be the route for SARS CoV-2 to enter the brain and the virus would reach the brain through vagal and/or splanchnic nerves (Esposito et al., 2020).

Severe SARS-CoV-2 infections have been found to be associated with neuropsychological effects and COVID-19 patients have been diagnosed with neuropathic pain including olfactory tract neuropathy, motor peripheral or axonal neuropathy, and sensory neuropathy (Abdelnour et al., 2020; Ghosh et al., 2020; Kirschenbaum et al., 2020; Li et al., 2021; Odriozola et al., 2021). The somatosensory nerve signals are sent to the brain from the spinal cord for further processing the sense of temperature, pressure, pain, touch, and vibration. Diseases impacting somatosensory nervous system such as diabetes, infections, nerve trauma, and autoimmune diseases can dysfunction the sensory signals into the spinal cord and the brain and lead to disorders associated with neuropathic pain (Campbell and Meyer, 2006). Neuropathic pain impairing the quality of life in patients reflects both central and peripheral sensitization and immune mechanism is involved in this matter of fact. Cooperation between the nervous system and the immune system have emerged as key in pain development (Ellis and Bennett, 2013). Proinflammatory cytokines and chemokines such as IL-1β and TNF-α construct the fundamental mechanism building the neuroimmune communication and lead to hyperalgesia and allodynia following nerve injury (Ellis and Bennett, 2013; Campbell and Meyer, 2006). Inhibition of increased chemokines and cytokines, and their receptors in central and peripheral nervous systems notably alleviates neuropathic pain. As mentioned earlier, the microbial composition plays a pivotal role in modulation of the immune system development, in addition to the pathogenesis of many inflammatory-related diseases (Rooks and Garrett, 2016). Amaral et al. (2008) explored the impact of microbiota on inflammatory pain with germ-free and conventional mice exposed to various inflammatory stimuli such as LPS, TNF-α, IL-1β, CXCL1, PGE2, and carrageenan. Their results demonstrated that commensal microbiota is required for mice to generate inflammatory hypernociception.

The gut microbiota is implicated in maintaining the function of the CNS via the immune system, endocrine system, or nervous system (Tognini, 2017; Chen et al., 2021). Previously, the contribution of the gut microbiota to the pathogenesis of the neuropathic pain accompanying cancer chemotherapy and inflammatory pain have been shown in studies using antibiotic-treated mice and germ-free mice (Amaral et al., 2008; Shen et al., 2017). Ding et al. investigated whether gut microbiota impact the neuropathic pain and if T-cell mediated immune responses are involved in this process using a mouse model of peripheral nerve injury-induced chronic neuropathic pain (Ding et al., 2021). Their results demonstrated that changes in the gut microbiota alter the development of neuropathic pain via shaping the balance between proinflammatory and antiinflammatory T cells. In another recent study using the same neuropathic pain model in rats, the relationship between the intestinal microbiota and related metabolites in neuropathic pain was explored (Chen et al., 2021). Gut microbiota, serum and spinal cord metabolomics, and pain-related parameters were compared to unravel their relation to each other. They reported that changes in the gut microbial composition was significantly correlated with the changes in the level of serum metabolites indicating that gut microbiota involved in modulating neuropathic pain and related metabolites, especially metabolites taking role in neuroinflammation signaling pathways, including arachidonic acid, beta-hydroxy butyric acid, 3-methylhistidine, 2-hydroxybutanoic acid, N6,N6,N6-trimethyl-l-lysine, l-histidine, l-anserine, l-tyrosine, dopamine, anthranilic acid, and kynurenic acid. 

Neurotransmitters, which can be either inflammatory or noninflammatory mediators, contribute to pain perception. GABA and glutamate are neurotransmitters commonly found in the body as inhibitory and excitatory neurotransmitters, respectively (Strandwitz et al., 2019). Both bacteria and host are capable of converting glutamate to GABA associated with regulating level of proinflammatory cytokines, modulating pain status, and maintaining gastrointestinal tract innervation. Human gut microbiota is predicted to comprise genera that are able to produce or consume GABA such as Escherichia, Bacteroides, and Parabacteroides species. GABA level has been reported to be modulated by altering the intestinal microbial composition, in particular Bacteroides which is negatively correlated with the brain signatures linked to depression (Strandwitz et al., 2019).

The interaction between the nervous system and the immune system is bidirectional; the nervous system, therefore, regulates innate and adaptive immunity and vice versa (Pitocco et al., 2021). The autonomic nervous system modulates the immune response via negative feedback process after the inflammatory cells deliver the sensory information. Once afferent neurons are activated, inflammatory mediators such as TNF-α can reach the CNS via circumventricular organs lacking blood-brain barrier. SARS-CoV-2 infection damages lungs with a hyperactive and imbalanced immune response (Li et al., 2020). Increased number of the proinflammatory cytokines in COVID-19 patients results in cytokine storm which is associated with severe COVID-19 and multiple organ dysfunction (Huang et al., 2020; Kalantar-Zadeh et al., 2020; Zheng et al., 2020). The gut–lung axis maintains host homeostasis and disease development with the association of immune system. The communication of the gut microbiota with immune system and lung influences the COVID-19 severity in patients with extrapulmonary conditions including neuropathy. SARS-CoV-2 infection may disturb the healthy intestinal microbiota and cause immune dysfunction and generalized inflammation (Aktas and Aslim, 2020). This dysbiosis may impair the gut permeability resulting in secondary infection with increased inflammation and affect distal organs via circulatory and lymphatic system. 

Moreover, gut microbiota plays a role in managing the tryptophan availability for kynurenine pathway involved in both central nervous and gastrointestinal system for inflammation regulation (Follmer, 2020; Zhang et al., 2021). Inflammation and tryptophan along with kynurenine pathway is thought to be linked to depression-like behavior with the association of dysbiosis and gut permeability (Leclercq et al., 2014). In addition, kynurenine was shown to induce pain hypersensitivity (Huang et al., 2016). Tryptophan serves as a precursor molecule to serotonin, kynurenine, and other downstream metabolites produced in the kynurenine pathway, and changes in the tryptophan availability can impact the ENS and CNS functionality in the gut–brain axis (O’Mahony et al., 2015; Kennedy et al., 2017). Inflammatory cytokines can induce the expression of the enzyme, indoleamine-2,3-dioxygenase (IDO), being involved in tryptophan catabolism through the kynurenine pathway and regulating hyperinflammatory responses (O’Mahony et al., 2015; Xiao et al., 2021). Tryptophan metabolism was shown to be altered in COVID-19 patients with an increased kynurenine:tryptophan ratio suggesting an elevated IDO activity (Shen et al., 2020; Thomas et al., 2020; Ansone et al., 2021). Recently, Xiao et al. (2021) studied integrated metabolites and cytokine analysis in COVID-19 patients and reported that kynurenine positively correlated with proinflammatory cytokines while tryptophan negatively correlated with those. Moreover, decrease in metabolites such as kynurenine represented the alleviation of the hyperactivation of tryptophan-kynurenine pathway. It has been demonstrated that plasma tryptophan concentration was increased in germ-free mice with decreased kynurenine:tryptophan ratio and the tryptophan level was normalized with colonization of the mice postweaning, suggesting the role of gut microbiota on tryptophan metabolism (Clarke et al., 2013). In another study feeding rats with Lactobacillus johnsonii, they found that level of serum kynurenine was significantly decreased in L. johnsonii fed rats compared to the controls (Valladares et al., 2013). Furthermore, plasma level of kynurenine was found to be increased in the patients with IBS relative to the controls and the IBS severity is correlated with kynurenine:tryptophan ratio (Fitzgerald et al., 2008; Gerard Clarke et al., 2009, 2012). These studies could indicate the possible link between the tryptophan metabolism and the gut–brain axis. Gut dysbiosis in COVID-19 patients may be the reason for the dysfunction of the kynurenine pathway which has been suggested to play an important role in oxidative stress and neuroinflammation in neurodegenerative diseases (Follmer, 2020). Significantly elevated levels of IL-6 and TNF-α are correlated with COVID-19 severity, and the concentration of IL-6 in COVID-19 patients is linked to the tryptophan metabolism into the kynurenine pathway (Zhang et al., 2021; Follmer, 2020). COVID-19 impairs the healthy gut microbiota and leads to immune dysfunction and generalized inflammation (Aktas and Aslim, 2020). A high level of peripheral cytokines crossing the blood-brain barrier induces the neuroinflammation which contributes to the pathophysiology of neurodegenerative diseases including neuropathies (Costello and Dalakas, 2020; Dolatshahi et al., 2021). On the other hand, various tissues and organs including respiratory and digestive tracts express ACE2 receptors, and corrupted ACE2 expression is thought to be related to viral infection, dysbiosis in gut microbiota, and immune imbalance (Chhibber-Goel et al., 2021). ACE2 was shown to maintain the neutral amino acid (tryptophan) transporter B0AT1 in the gut and coordinate antimicrobial peptides which helps gut stability (Hashimoto et al., 2012). Diminished tryptophan level due to impaired ACE2 expression fails to induce the secretion of antimicrobial peptides which then leads to pathogen survival and dysbiosis in the gut (Chhibber-Goel et al., 2021; Rajput et al., 2021). Since this dysbiosis subsequently results in susceptibility to the intestinal inflammation, the COVID-19 patients with GI complications may have increased respiratory distress relative to the patients with no gut involvement (Chhibber-Goel et al., 2021; Rajput et al., 2021; Zhang et al., 2021).

## 5. Gut microbiota targeted interventions in COVID-19

Taken together all these interactions between the gut microbiota, the immune system, and the distal organs including lung and brain, gut microbial dysbiosis could be the reason of the neurologic complications seen in severe COVID-19 patients with the association of dysbiosis-related neuroinflammation. SARS-CoV-2 affects brain functions most likely through the gut–brain axis and induces neuropathy associated with dysbiosis-related inflammation.

Targeted microbial alteration in the GI system could be a strategy to attenuate the disease symptoms in COVID-19 patients. Fecal microbiota transplantation (FMT) performed to treat patients with Clostridium difficile infection is an excellent example of maintaining health and disease through microbiota modification (Bakken et al., 2011). FMT is an effective powerful way of modulating the disrupted intestinal microbiota and improving immunity and could be considered an alternative approach to reduce neuropathy in COVID-19 patients and the disease severity. Diabetic neuropathy is thought to play a role in COVID-19 severity with dysfunction of the autonomic nervous system determining the failure in regulation of immune response and leading to decrease in pulmonary function (Pitocco et al., 2021). FMT has been promoted as an alternative strategy to relieve diabetic neuropathy in a patient with history of diabetes and hypertension for 8 years (Cai et al., 2018). Recently, FMT procedure has been applied to improve chronic norovirus infection in an immunosuppressed patient with kidney transplant (Barberio et al., 2020). After 5 days of FMT, the stool sample was negative for norovirus infection with complete symptom resolution. FMT has been evaluated as a potential strategy to manage viral hepatitis as well^2^
ClinicalTrials.gov. (2021). ClinicalTrials. https://clinicaltrials.gov/ (Sehgal et al., 2020). There is one clinical trial published on FMT application against COVID-19 with a few limitations such as small sample size (Liu et al., 2021). The study was performed on patients cured and discharged from hospital instead of COVID-19 patients under treatment in hospital and the trial was nonrandomized. They reported that FMT restored the gut microbiota by increasing the abundance of Faecalibacterium and Bifidobacterium. Probiotics are another approach for targeted microbial modulation in the gastrointestinal system that could help with improving COVID-19 symptoms. Experimental and clinical studies exploring the impact of probiotics on respiratory system viruses, such as influenza, to improve the severity of viral respiratory tract infections and to decrease the risk of infection have been documented well (Singh and Rao, 2021). Clinical trials2 all around the world are ongoing to test the impact of probiotics on modulation of the gut microbiota and the efficacy in patients with COVID-19 patients with only two of them published yet. In a trial comprising 70 individuals, SARS-CoV-2–positive patients received either only the required drug therapy or a commercial multistrain probiotic formulation along with the drug therapy during hospitalization (d’Ettorre et al., 2020). The probiotic supplementation showed a reduction in the severity of COVID-19 patients with improvement in diarrhea and other COVID-19–associated symptoms within about 72 h. Moreover, risk of developing respiratory failure and the ratio of the patients moved to ICU were higher in the nonprobiotic group relative to the probiotic group. Another trial with the same probiotic formulation delivered to 200 patients with severe COVID-19 pneumonia, the mortality rate, the ICU hospitalization emergency, and the length of hospitalization were evaluated (Ceccarelli et al., 2021). The mortality rate was much higher in the nonprobiotic group with 30% compared to the probiotic group with 11%. 

## 6. Conclusion

It is well established that gut microbiota contributes to the COVID-19 severity with the association of the gut–lung axis (Ferreira et al., 2020; Jin et al., 2020; Wan et al., 2020; Tao et al., 2020). Gut microbiota serves as an extra organ and maintains physiological and homeostatic functions by communicating distal organs with the association of immunomodulation (Dachuan et al., 2019; Dapeng et al., 2020). The gut–brain axis is another crosstalk that gut microbiota is involved in COVID-19 manifestations and severity of the disease. This axis is linked to various tissues and organs, including immune cells, autonomic nervous systems, glands, brain, and intestine, with its microbial composition and communicates with each to preserve homeostasis. Recent studies support that gut microbiota plays a crucial role in neuropathic pain, abdominal pain, headache, and inflammatory pain (Amaral et al., 2008; Guo et al., 2019; Cuozzo et al., 2021). Dysbiosis of the gut microbiota, either at the onset of SARS-CoV-2 infection or during the course of the disease with drug treatment, could be the reason for COVID-19 manifestations, including neuropathic pain, abdominal pain, headache and more. Although there has been no effective drug or treatment for COVID-19, a variety of treatment regimens have been applied to the patients infected by SARS-CoV-2. Different therapeutic interventions can result in dysbiosis and eventually impact the brain through the gut–brain axis with drug-associated dysbiosis. Regardless of the order of the incidents leading to dysbiosis in patients with COVID-19, alteration in the gut microbiota tends to influence the bidirectional relationship between the gut and the brain. Considering the relationship between the gut microbiota and the immune system influencing the neuropathy in COVID-19 patients, rebuilding the gut microbiota to maintain a healthy status could be an alternative strategy to improve the neuropathy in COVID-19 patients. Specific interventions such as live biotherapeutics or microbiota-derived metabolites-based approaches might be effective in COVID-19 patients for dysbiosis-associated neuropathy.
